# Effects of endurance training on reduction of plasma glucose during high intensity constant and incremental speed tests in Wistar rats

**DOI:** 10.1590/1414-431X20165226

**Published:** 2016-10-24

**Authors:** P. Abreu, K.F. Vitzel, I.C.C.R. Monteiro, T.I. Lima, A.N. Queiroz, J.H. Leal-Cardoso, S.M. Hirabara, V.M. Ceccatto

**Affiliations:** 1 Departamento de Fisiologia e Biofísica, Instituto de Ciências Biomédicas, Universidade de São Paulo, São Paulo, SP, Brasil; 2 Instituto de Biofísica Carlos Chagas Filho, Universidade Federal do Rio de Janeiro, Rio de Janeiro, RJ, Brasil; 3 Faculdade de Medicina de Ribeirão Preto, Universidade de São Paulo, Ribeirão Preto, SP, Brasil; 4 Hospital e Maternidade José Martiniano de Alencar, Fortaleza, CE, Brasil; 5 Instituto Superior de Ciências Biomédicas, Universidade Estadual do Ceará, Fortaleza, CE, Brasil; 6 Instituto de Ciências da Atividade Física e Esporte, Universidade Cruzeiro do Sul, São Paulo, SP, Brasil

**Keywords:** Exercise, skeletal muscle, Liver, Blood glucose, Training

## Abstract

The aim of this research was to investigate the effects of endurance training on reduction of plasma glucose during high intensity constant and incremental speed tests in Wistar rats. We hypothesized that plasma glucose might be decreased in the exercised group during heavy (more intense) exercise. Twenty-four 10-week-old male Wistar rats were randomly assigned to sedentary and exercised groups. The prescription of endurance exercise training intensity was determined as 60% of the maximum intensity reached at the incremental speed test. The animals were trained by running on a motorized treadmill, five days/week for a total period of 67 weeks. Plasma glucose during the constant speed test in the exercised group at 20 m/min was reduced at the 14th, 21st and 28th min compared to the sedentary group, as well at 25 m/min at the 21st and 28th min. Plasma glucose during the incremental speed test was decreased in the exercised group at the moment of exhaustion (48th min) compared to the sedentary group (27th min). Endurance training positively modulates the mitochondrial activity and capacity of substrate oxidation in muscle and liver. Thus, in contrast to other studies on high load of exercise, the effects of endurance training on the decrease of plasma glucose during constant and incremental speed tests was significantly higher in exercised than in sedentary rats and associated with improved muscle and hepatic oxidative capacity, constituting an important non-pharmacological intervention tool for the prevention of insulin resistance, including type 2 diabetes mellitus.

## Introduction

Chronic endurance exercise leads to a shift in fuel metabolism from carbohydrates to fat, in part due to an increase in the expression of peroxisome proliferator-activated receptor gamma co-activator 1-alpha (PGC-1α), which plays a central role in the regulation of mitochondrial biogenesis and participates in the regulation of both carbohydrate and lipid metabolism in skeletal muscle. Additionally, PGC-1α promotes the remodeling of muscle tissue to a fiber-type composition that is metabolically more oxidative (1). The pyruvate dehydrogenase kinase 4 (PDK4) transcription is markedly increased in skeletal muscle during prolonged endurance exercise training and after short-term high-intensity exercise. PDK4 expression is also increased during recovery periods, suggesting an essential role of this protein in the replenishment of muscle glycogen store and in the reestablishment of intracellular homeostasis after exercise, mainly by preventing carbohydrate oxidation and by greatly stimulating fatty acid oxidation (2).

The training-induced decrease in glucose production is due to alterations in the glucoregulatory hormone in liver (hepatic glycogenolysis and gluconeogenesis) (3). The increase of citrate synthase activity and of cytochrome C (Cyt C) have been utilized as markers of mitochondrial oxidative phosphorylation (3). It has been previously observed that a single bout of exercise increases phosphorylated acetyl-CoA carboxylase (p-ACC) in the liver of mice and rats (4). These findings indicate that exercise affects the hepatic metabolism of carbohydrates and fatty acids, driving glucose metabolism towards fatty acid oxidation. In fact, it was demonstrated that phosphorylation of ACC in the liver results in decreased synthesis and increased oxidation of fatty acids (4).

The reduction in glucose utilization during exercise and after training is in part due to a decrease in muscle glucose uptake (5,6). This has primarily been shown when the same absolute workload is performed in the sedentary and exercised states whereas, when work is performed at the same relative intensity, the difference in fuel utilization is generally smaller or nonexistent (5,6). By analogy, it might be expected that the training-induced increase in muscle transport of glucose would result in greater, not reduced, utilization of glucose during exercise. However, evidence suggests that, during submaximal exercise at the same absolute intensity, glucose utilization is decreased in the exercised state (7).

Thus, training is usually associated with decreased plasma glucose uptake in exercised compared with sedentary state (8,9). Therefore, at maximal or near-maximal intensities during constant and incremental speed tests, high metabolic protein content in exercised muscle is important for enhancing glucose uptake. Possible contributions of the metabolic alterations in the skeletal muscle and liver induced by endurance exercise in attenuating pathological conditions and diseases are currently discussed in the literature.

The aim of this study was to investigate the effects of endurance training on the reduction of plasma glucose during high intensity constant and incremental speed tests in exercised Wistar rats. This was performed during conditions when maximal exercise-induced muscle glucose uptake is expected, i.e., during exercise performed at a high intensity in association with the muscle and hepatic oxidative capacity.

## Material and Methods

### Animals

Twenty-four male Wistar rats (10 weeks of age, body mass of 200–230 g), provided by the Animal Facility of the Universidade Estadual do Ceará, were used in the experiments. The animals were randomly separated into two groups: sedentary and exercised. The animals were maintained (5 rats per cage) on a 12:12 h light-dark cycle, in a room with controlled temperature, and were given *ad libitum* access to food and water. The principles of Laboratory Animal Care were followed, and all experiments were approved by the Institutional Animal Care and Use Committee of the Universidade Estadual do Ceará, Brazil (Protocol #10030319-6). All experimental procedures were performed during the dark cycle.

### Exercise training protocol

The prescription of endurance exercise training intensity was determined as 60% of the maximum intensity reached at the incremental speed test, as previously described by Abreu et al. (10). The training intensity was changed over time as the physical capacity of the rats increased. The animals were trained by running on a motorized treadmill (Athletic Speed 2, Athletic, Brazil), 5 days/week for a total period of 6 weeks. Thirty-six hours after the last exercise session, the animals were anesthetized with ketamine (80 mg/kg) and xylazine (12 mg/kg) and sacrificed by decapitation. The fat depots, skeletal muscles, and liver were quickly removed, immediately frozen in liquid nitrogen and stored at −80°C until the proper procedures were performed, according to the analysis protocols described below.

### Constant and incremental speed test protocols

The treadmill used in the present study was not equipped with electric grids at the rear of the treadmill lanes to provide an aversive stimulus to keep the animals exercising. Instead, a non-painful manual stimulus was sufficient to motivate the rats to continue running on the treadmill. The test protocol was finished when the animal reached exhaustion, defined as the refusal or incapacity to run even under manual prodding, or when the coordination between the anterior and posterior paws became impaired (10). After endurance exercise training protocol of 6 weeks, the rats were submitted to constant speed test (velocities of 15, 20, and 25 m/min, during 28 min) and incremental speed test (a series of 3 min running steps, with increments of 3.3 m/min between the subsequent steps, at 0% inclination). Additionally, after an experimental training period of the constant and incremental speed test protocol, blood samples (10 µL) were taken from the caudal vein at the start and every 7 min of constant speed test and every 3 min of incremental speed test, for blood glucose measurement (Accu-Chek Active Kit, Roche, USA).

### Citrate synthase (CS) activity

CS activity was measured as previously described by Srere (11). The red and white portions of the gastrocnemius (RG, and WG, respectively) and soleus (S) muscles, as well as the liver (L) were analyzed by enzymatic colorimetric assay performed in a spectrophotometer.

### Myosin ATPase activity

Myosin ATPase activity was determined as previously described by Simonides and van Hardeveld (12). Frozen gastrocnemius muscle was cut into cross sections using a cryostat (Micron HM505E; Zeiss, Germany). The myosin ATPase reaction was used to identify the muscle fiber type I or II and the images were analyzed using the Image-Pro Plus software (Media Cybernetics, USA), using an optical microscope (Nikon Eclipse E1000, Japan) with a coupled camera (Nixkon DXM 1200).

### Glycogen content

Glycogen content was evaluated as previously described by Dreiling et al. (13). The samples were read by a colorimetric method in a spectrophotometer (Glucose Pap Liquiform Labtest, Brazil).

### Western blotting analysis

Content of specific proteins was studied by western blotting analysis as previously described by Towbin et al. (14). The RG, WG and S muscles, and liver tissues were homogenized in a lysis buffer. The primary antibodies utilized were peroxisome proliferator-activated receptor gamma coactivator 1-alpha (PGC-1α; ab106814; 91 kDa), pyruvate dehydrogenase kinase 4 (PDK-4; ab89295; 46 kDa), phospho-acetyl-CoA carboxylase (p-ACC Ser79; SAB4503799; 265 kDa) and cytochrome C (Cyt C; ab133504; 11 kDa). Equal protein loading was confirmed by Ponceau S staining of all membranes (15).

### Statistical analysis

Data are reported as means±SE and were analyzed by using GraphPad Prism software (4.0, GraphPad Inc., USA). One-way ANOVA followed by Tukey’s multiple comparison *post hoc* test were used to assess differences among groups; for comparison between two groups, the Student’s *t*-test was used. Differences were considered to be statistically significant at P<0.05.

## Results

As shown in Table 1, endurance training resulted in a pronounced reduction of retroperitoneal and epididymal fat mass, an increase of CS activity in RG and WG, S and L, a rise of type I fiber proportion in gastrocnemius muscle, measured by ATPase activity (Supplementary Figure S1), and an elevation of glycogen content in L (Table 1). There was no change in body mass of animals during the period of endurance training.

**Table:**
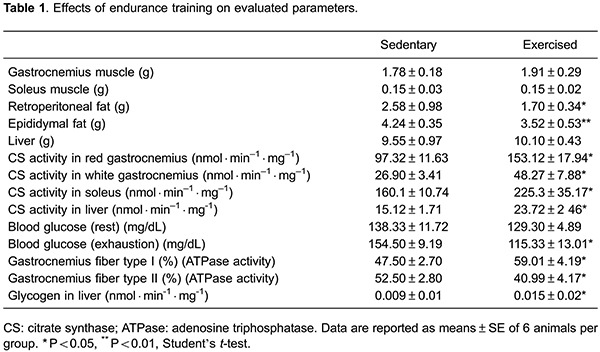

### Effects of endurance training on plasma glucose during constant and incremental speed tests

**Figure 1:**
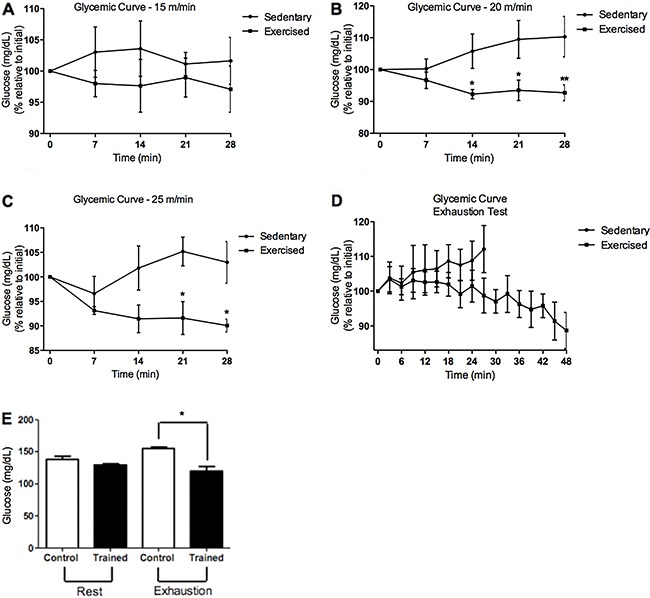
Effects of endurance training on blood glucose during constant (*A, B* and *C*) and incremental speed test. During the incremental speed test, the sedentary group achieved exhaustion at the 27th min, while the exercised group was exhausted only at the 48th min (*D*) with lower blood glucose levels (*E*). Data are reported as means±SE of 6 animals per group. *P<0.05, **P<0.01, one-way ANOVA followed by the Tukey’s multiple comparison *post hoc* test was used to assess differences among groups and Student’s *t*-test for comparison between two groups.

There was no alteration on blood glucose content during the constant speed test at 15 m/min (Figure 1A). At 20 m/min, the blood glucose was reduced at the 14th, 21st, and 28th min in the exercised group when compared to the sedentary group (Figure 1B), as well as at 25 m/min at the 21st and 28th min (Figure 1C). During the incremental speed test, the sedentary group achieved exhaustion at the 27th min, while the exercised group was exhausted only at the 48th min (Figure 1D). Blood glucose was lower in the exercised group at the moment of exhaustion when compared to sedentary group (Figure 1E).

### Effects of endurance training on PGC-1α, PDK4, p-ACC and Cyt C

**Figure 2:**
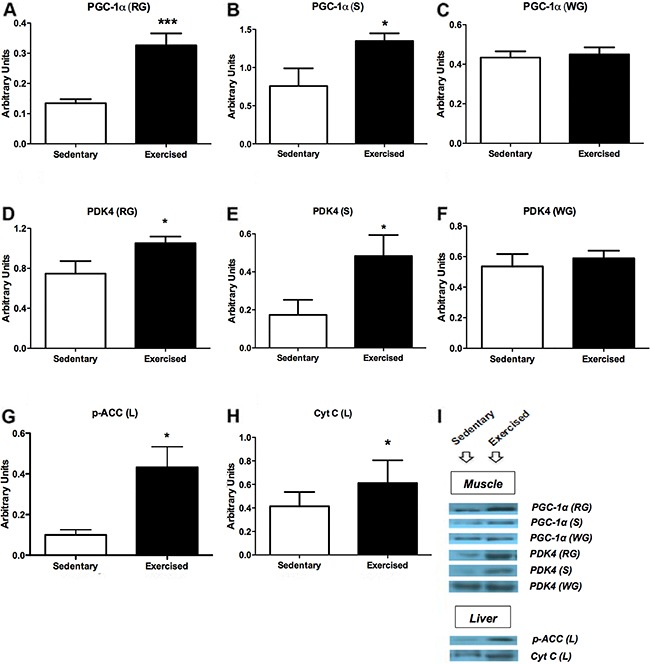
Quantification by western blotting of peroxisome proliferator-activated receptor gamma coactivator 1-alpha (PGC-1α) in red gastrocnemius (RG) (*A*), soleus (S) (*B*) and white gastrocnemius muscles (WG) (*C*). Pyruvate dehydrogenase kinase-4 (PDK4) content in RG (*D*), S (*E*) and WG (*F*). Phospho-acetyl-CoA carboxylase (p-ACC Ser79) content in liver (L) (*G*). Cytochrome C (Cyt C) in L (*H*). Representative blots of each evaluated protein (*I*). Data are reported as means±SE of 6 animals per group. *P<0.05, ***P<0.01, Student’s *t*-test.

Endurance training resulted in increased PGC-1α in RG (2.4-fold; Figure 2A) and S (78%; Figure 2B), and PDK4 in RG (43%; Figure 2D) and S (2.7 fold; Figure 2E). No effect was observed in the WG (Figure 2C and F). The endurance training also raised the hepatic p-ACC (Ser79; 4.2 fold; Figure 2G) and Cyt C (2.4 fold; Figure 2H). Equal protein loading was confirmed by Ponceau S staining of all membranes (Supplementary Figure S2).

## Discussion

In previous studies (5,6), it has been demonstrated that exercise or exercised state greatly increase the plasma glucose concentration and consequently decrease the glucose uptake in human and rodent models during physical exercise session at moderate intensity. A reduction in glucose utilization by skeletal muscle occurs when exercise is performed at the same intensity compared to the sedentary group or to the endurance training pre-program (before starting the endurance training program). Here, we demonstrated that endurance exercise training promoted important adaptations in skeletal muscle by increasing protein content of PGC-1α and PDK-4, and enzyme activity of citrate synthase and myosin ATPase. Also, in the liver, elevated citrate synthase activity and cytochrome C content, proteins related to mitochondrial oxidative capacity and phosphorylation processes, were observed, and elevated acetyl-CoA carboxylase phosphorylation, suggesting a reduced *de novo* lipogenesis. These effects were directly related to the decreased plasma glucose levels during high-intensity exercise (constant and incremental speed test protocols). Previous studies have suggested an increase in glucose utilization after endurance training protocol (16,17); however, this is the first study to demonstrate a direct interaction among exercise intensity, glucose utilization, and muscle and hepatic oxidative capacity.

The regulatory function of metabolic proteins observed in lipid metabolism in endurance exercise is of particular interest, since it can be an interesting target for pharmacological intervention in the prevention or therapy of systemic disorders related to energy metabolism.

In the present study, we demonstrated that endurance training protocol promoted an up regulation of PGC-1α protein content in skeletal muscle. This protein has been related to the stimulation of mitochondrial biogenesis and remodeling of muscle tissue towards an oxidative fiber-type profile as depicted by the increase proportion of type I fibers in the gastrocnemius muscle after training (1). Also, the up regulation of PDK4 in skeletal muscle by chronic endurance exercise indicates a potential inhibition of carbohydrate oxidation, which could contribute for the reestablishment and/or sparing of the muscle glycogen stores and intracellular homeostasis after exercise (2).

As found in a previous study, liver from exercised rats presents reduced gluconeogenesis and glycogenolysis after exercise (4). In our study, we found that these hepatic metabolic improvements are related to increased citrate synthase activity and Cyt C, proteins related to mitochondrial oxidative capacity and phosphorylation processes. Furthermore, we found an increase in the p-ACC, an indicator of the inhibition of *de novo* hepatic lipogenesis. The phosphorylation of ACC has been related to the decreased expression of lipogenic proteins and elevated lipid oxidation in liver of healthy animals (4). Collectively, our findings demonstrate important contributions of regular physical activity in altering energetic metabolism in liver. Further mechanistic studies are required to determine the effect of exercise-mediated peripheral changes (liver) on systemic metabolic alterations.

Previously, it was demonstrated that exercise rises the level of GLUT-4 protein in skeletal muscle related to an increase of contraction-stimulated glucose transport in rat muscle. However, in this study, exercise did not cause a constant rise in muscle glucose uptake and the contraction-induced glucose transport were similar in trained and untrained muscles (18). Interestingly, Kawanaka et al. (19) investigated the relationship between the quantity of tetanic contractions and the rise in glucose transport in exercised and sedentary muscle. They observed that a great number of tetanic contractions are needed to maximally activate muscle glucose transport in exercised rats when compared to sedentary rats, suggesting that the increased glycogen level induced by training may inhibit glucose transport (19).

Thus, in contrast with previous studies (5,6), we observed that endurance training exercise and increased muscle oxidative capacity promoted an elevated transport and utilization of glucose, decreasing the level of plasma glucose during high-intensity exercise session, in association with improved mitochondrial oxidative capacity and phosphorylation in liver. We also observed that muscle substrate utilization during exercise was influenced by several intrinsic exercise-related factors including exercise intensity, metabolic factors (enzyme activities), glucose delivery rate and cycle activity, and muscle-liver interaction.

Thus, adaptation to endurance training including muscle and hepatic oxidative metabolic alterations can participate in the reduction of plasma glucose during high-intensity constant and incremental speed tests. These effects can help to explain, at least in part, the increased glucose utilization and reversal of fatigue in athletes with low glycogen stores during prolonged exercise sessions, helping to maintain the contraction-stimulated maximal glucose transport and utilization in skeletal muscle (20). Accordingly, previous studies have found that high-intensity exercise increases insulin sensitivity and oxidative capacity in skeletal muscle and liver (4, 16-19).

In conclusion, our findings support the benefits of physical endurance exercise in improving glucose homeostasis and insulin sensibility, constituting an important non-pharmacological intervention tool for the prevention or therapy of insulin-resistance, including obesity, metabolic syndrome, dyslipidemias, and type 2 diabetes mellitus.

## Supplementary material

Click here to view [pdf].
